# YbdO Promotes the Pathogenicity of *Escherichia coli* K1 by Regulating Capsule Synthesis

**DOI:** 10.3390/ijms23105543

**Published:** 2022-05-16

**Authors:** Yu Fan, Hongmin Sun, Wen Yang, Jing Bai, Peng Liu, Min Huang, Xi Guo, Bin Yang, Lu Feng

**Affiliations:** 1 The Key Laboratory of Molecular Microbiology and Technology, Ministry of Education, Nankai University, Tianjin 300457, China; 1120170077@mail.nankai.edu.cn (Y.F.); sun13820589167@163.com (H.S.); yangwen@mail.nankai.edu.cn (W.Y.); bj1551880084@163.com (J.B.); 1120170078@mail.nankai.edu.cn (P.L.); huangmin0304@163.com (M.H.); guoxi@nankai.edu.cn (X.G.); 2 Tianjin Key Laboratory of Microbial Functional Genomics, TEDA Institute of Biological Sciences and Biotechnology, Nankai University, Tianjin 300457, China

**Keywords:** *E. coli* K1, *ybdO*, H-NS, pH, K1 capsule, HBMECs, neonatal meningitis

## Abstract

*Escherichia coli* K1 is the most popular neonatal meningitis-causing Gram-negative bacterium. As a key virulence determinant, the K1 capsule enhances the survival of *E. coli* K1 in human brain microvascular endothelial cells (HBMECs) upon crossing the blood–brain barrier; however, the regulatory mechanisms of capsule synthesis during *E. coli* K1 invasion of HBMECs remain unclear. Here, we identified YbdO as a transcriptional regulator that promotes *E. coli* K1 invasion of HBMECs by directly activating K1 capsule gene expression to increase K1 capsule synthesis. We found that *ybdO* deletion significantly reduced HBMEC invasion by *E. coli* K1 and meningitis occurrence in mice. Additionally, electrophoretic mobility shift assay and chromatin immunoprecipitation–quantitative polymerase chain reaction analysis indicated that YbdO directly activates *kpsMT* and *neuDBACES* expression, which encode products involved in K1 capsule transport and synthesis by directly binding to the *kpsM* promoter. Furthermore, *ybdO* transcription was directly repressed by histone-like nucleoid structuring protein (H-NS), and we observed that acidic pH similar to that of early and late endosomes relieves this transcriptional repression. These findings demonstrated the regulatory mechanism of YbdO on K1 capsule synthesis, providing further insights into the evolution of *E. coli* K1 pathogenesis and host–pathogen interaction.

## 1. Introduction

Newborn bacterial meningitis is an acute inflammation of the meninges, subarachnoid space, and brain vasculature caused by bacteria or bacterial products and associated with substantial mortality and morbidity worldwide [1,2,3,4]. As defined by organism isolation from cerebrospinal fluid (CSF) cultures, *Escherichia coli* K1 is the most common Gram-negative bacterium that causes meningitis in newborns [5] and represents ~80% of the CSF isolates identified in meningitic neonates [6]. The molecular mechanisms involved in the pathogenesis of *E. coli* K1 leading to this severe disease are not fully understood [7].

Most cases of *E. coli*-caused meningitis occur as a consequence of hematogenous spread [8], and circulating *E. coli* enters the brain parenchyma by penetrating the blood–brain barrier (BBB), which is the most critical pathogenic step [9]. Several studies in humans and experimental animals suggest that high-degree bacteremia (e.g., >10^5^ CFU/mL of neonatal animal blood [10]; >10^3^ CFU/mL of neonatal infant blood [11]) is a prerequisite for meningeal invasion based on its enabling *E. coli* K1 escape from host defenses to cause meningitis. Penetration of the brain by *E. coli* K1 involves its binding to and invasion of human brain microvascular endothelial cells (HBMECs) that constitute the BBB. *E. coli* K1 binds to HBMECs by interacting with CD48 and sialoglycoproteins through type 1 fimbria [12,13] and S fimbria [14,15,16], respectively. *E. coli* K1 invades HBMECs through the interactions of cytotoxic necrotizing factor 1 (CNF1) [17,18], outer membrane protein A (OmpA) [19,20], and the invasion protein IbeA [21,22,23] with the host proteins 37LRP, GP96, and Caspr1, respectively. The K1 capsule contributes to high-level bacteremia and allows *E. coli* K1 to survive intracellularly and traverse HBMECs as live bacteria [8,10,24,25]. Studies have identified several molecules involved in regulating *E. coli* K1 virulence, including RpoE and RfaH, which positively regulate the expression of *ompA* and *cnf1*, respectively [26,27]; however, the regulatory mechanism of the virulence factors remains unclear in *E. coli* K1.

The K1 capsule, which is important for reducing immunogenicity by mimicking host antigens and avoiding serum killing and complement-mediated opsonophagocytosis in the host, is a key determinant of *E. coli* K1 virulence [10,28]. Previous studies demonstrate that K1 capsule expression is essential for *E. coli* survival in HBMECs and that *neuDB* mutations prevent the formation of the K1 capsule and impair bacterial viability during BBB invasion [8]. Specifically, the K1 capsule is responsible for maintaining bacterial viability during BBB invasion, and a previous study showed that the recovery of viable intracellular organisms of the K1+ strain from HBMECs was significantly higher than that of the K1 strain [24].

Biosynthesis and assembly of capsular polysaccharides are complex processes. The K1 capsule gene cluster comprises three regions (1, 2, and 3). Regions 1 (*kpsFEDUCS*) and 3 (*kpsMT*) are conserved and encode proteins involved in translocating polysaccharides from the cytoplasmic site of synthesis through the two membranes of the Gram-negative cell envelope to the cell surface [29,30]; region 2 (*neuDBACES*) is serotype-specific and encodes several enzymes involved in the biosynthesis and activation of sialic acid in the K1 capsule [29,30]. Regions 2 and 3 on the same transcriptional unit share the *kpsM* promoter, whereas region 1 is organized in a single transcriptional unit, with the promoter located upstream of *kpsF*. The genes in the K1 capsule operons are mainly regulated at a transcriptional level, and several global regulators (FNR, H-NS, SlyA, IHF, and RfaH) exert their regulatory function by directly binding to the promoters of region 1 or 3 [31,32,33,34]. However, none of these was reported to regulate capsule synthesis during *E. coli* K1 infection of HBMECs.

*ybdO* encodes a putative transcriptional regulator in *E. coli* K1. Domain structure analysis revealed that the *ybdO* gene product contains an N-terminal DNA-binding helix-turn-helix (HTH) motif and a C-terminal co-factor-binding domain (Figure S1), which are conserved in the LysR-type transcriptional regulator (LTTR) family. LTTRs regulate a variety of genes, including those involved in virulence, metabolism, quorum sensing, and motility [35]. *ybdO* is a horizontally-transferred gene that may have facilitated the adaptation of *E. coli* K1 to new ecological niches [36]. However, the full contribution of YbdO to *E. coli* K1 virulence and pathogenicity remains largely unknown.

In this study, we investigated the contribution of *ybdO* to *E. coli* K1 virulence by combining comparative transcriptome analysis and infection modes both in vitro and in vivo. The results showed that YbdO promoted the invasion of HBMECs by *E. coli* K1 by increasing K1 capsule production. Furthermore, during *E. coli* K1 invasion, histone-like nucleoid structuring protein (H-NS) senses the acidic pH within endosomes to de-repress *ybdO* transcription, resulting in increased YbdO-dependent capsule synthesis.

## 2. Results

### 2.1. E. coli K1 Invasion of HBMECs Increases ybdO Expression

To investigate the regulatory mechanisms associated with *E. coli* K1 invasion, we performed transcriptome analysis to reveal differences in the gene-expression profile between *E. coli* K1 cultured in brain–heart infusion (BHI) media alone and when invading HBMECs. We identified downregulation and upregulation of the expression of 1335 and 1359 genes, respectively, in HBMEC-invading *E. coli* K1 relative to BHI-cultivated *E. coli* K1 (Table S3). Classification of the differentially upregulated genes using Kyoto Encyclopedia of Genes and Genomes (KEGG) analysis revealed significant enrichment of the KEGG categories ABC transporters, two-component systems, and propanoate metabolism (Figure S2). KEGG categories that were significantly enriched for the downregulated genes mainly included cofactor biosynthesis, amino sugar and nucleotide sugar metabolism, and oxidative phosphorylation (Figure S2).

**Figure 1:**
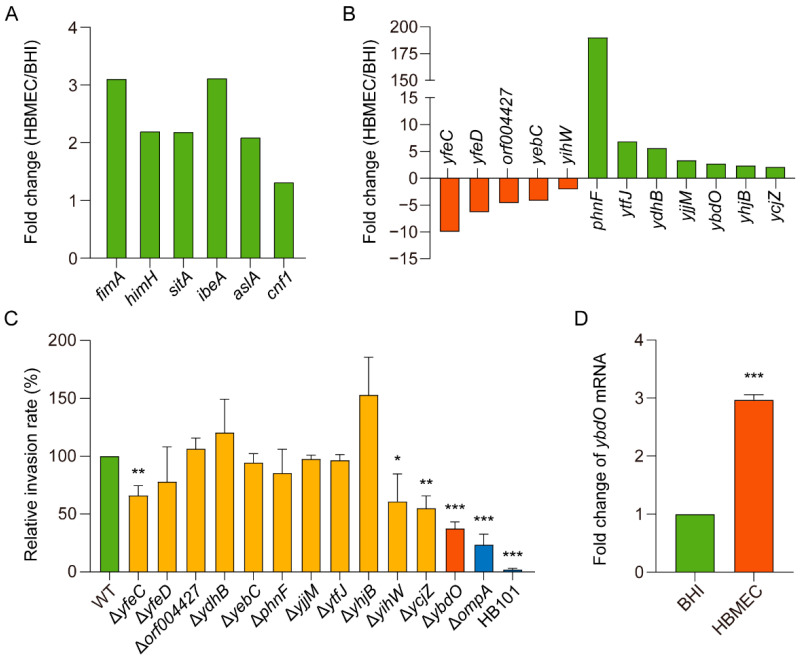
Significant upregulation of *ybdO* expression following *E. coli* K1 invasion of HBMECs. (**A**) Fold change in the fragments per kilobase of transcript per million fragments mapped (FPKM) of representative differentially expressed toxic genes. (**B**) Fold change in the FPKM of putative transcriptional regulators. (**C**) Mutation of putative transcriptional regulators revealed their different roles in HBMEC invasion. (**D**) To validate RNA-seq results, *ybdO* expression was analyzed by qRT-PCR. Data were obtained from three independent experiments and analyzed using Student’s *t*-test. * *p* < 0.05, ** *p* < 0.01, *** *p* < 0.001.

Interestingly, many known virulence genes in *E. coli* K1 were upregulated, including genes encoding type 1 and S fimbriae, which are associated with HBMEC binding, and *cnf1*, *ibeA*, *aslA*, and *sitA*, which are associated with HBMEC invasion (Figure 1A) [37]. These results identified substantially upregulated expression of virulence factors upon *E. coli* K1 invasion of HBMECs, and that various transcription factors (TFs) likely mediate their expression.

Comparative transcriptome analysis revealed significant changes in the expression of twelve genes encoding putative transcriptional regulators possibly involved in virulence regulation (Figure 1B). To determine the roles of these putative TFs in *E. coli* K1 invasion of HBMECs, we constructed isogenic in-frame-deletion mutants of potential TFs. For the invasion assay, we chose the Δ*ompA* mutant strain and the non-invasion strain HB101 as a positive control for decreased invasion and negative control, respectively [38]. Among these TF mutants, the invasion rate of four (Δ*yfeC*, Δ*yihW*, Δ*ycjZ*, and Δ*ybdO*) significantly decreased, with that of the Δ*ybdO* mutant decreasing the most (Figure 1C). Quantitative reverse transcription polymerase chain reaction (qRT-PCR) analysis revealed a 2.97-fold increase in *ybdO* expression during HBMEC invasion as compared with that observed in BHI cultures (Figure 1D), which agreed with the RNA-seq results. These findings suggested that YbdO might play an important regulatory role in *E. coli* K1 invasion of HBMECs.

### 2.2. YbdO Contributes to E. coli K1 Invasion of HBMECs

**Figure 2:**
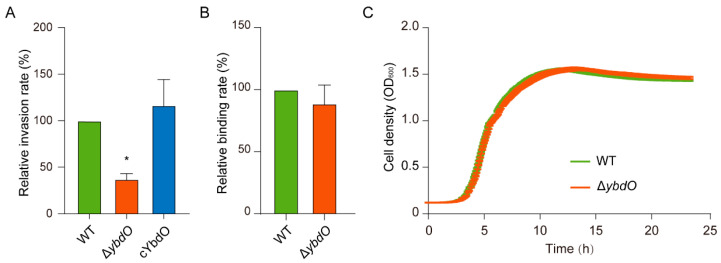
*ybdO* deletion reduces HBMEC invasion by *E. coli* K1. (**A**) Differences in the invasion abilities of the Δ*ybdO* and *cybdO* strains relative to that of the WT strain. (**B**) Differences in the binding abilities of the Δ*ybdO* and *cybdO* strains relative to that of the WT strain. (**C**) Growth of WT and Δ*ybdO* strains in BHI medium. Data were obtained from three independent experiments and analyzed using Student’s *t*-test. * *p* < 0.05.

To determine whether YbdO affects HBMEC invasion by *E. coli* K1, we constructed a complementary strain (*cybdO*) for virulence evaluation. We found that the invasion rate of the Δ*ybdO* mutant, as indicated by the number of bacteria entering HBMECs, was reduced by 2.90-fold as compared with that observed in the wild-type (WT) strain (Figure 2A), whereas the invasion rate of the complementary strain (*cybdO*) was comparable with that of the WT strain (Figure 2A). Additionally, the growth rate of the Δ*ybdO* mutant in BHI broth was the same as that of the WT strain (Figure 2C), demonstrating that the decreased invasion rate was not due to different growth rates. These results indicated that YbdO promotes the invasion of HBMECs by *E. coli* K1. Because binding occurs before invasion, we examined the ability of the Δ*ybdO* mutant strain to bind to HBMECs, finding that the binding rate was comparable with that of the WT strain (Figure 2B), indicating that YbdO does not affect *E. coli* K1 binding to HBMECs. Consistent with the lack of effect on binding, *ybdO* expression did not change upon HBMEC binding as compared with that of the mutant grown in BHI medium (Figure S3).

### 2.3. YbdO Promotes Meningitis in Mice

**Figure 3:**
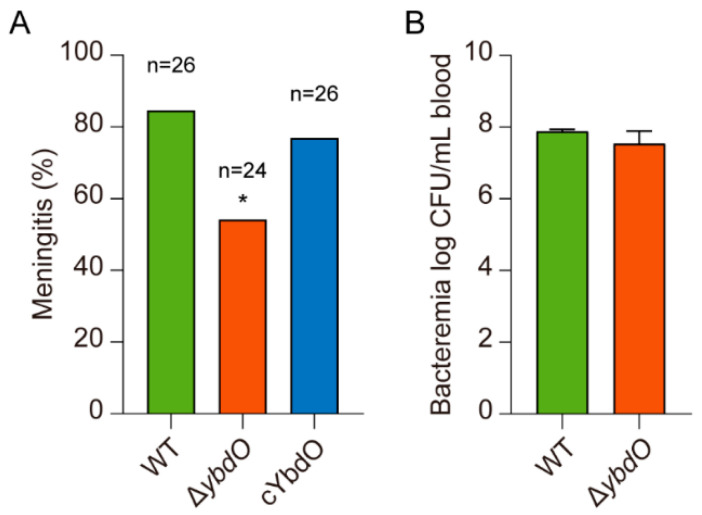
YbdO promotes *E. coli* K1-mediated meningitis without changes in high-level bacteremia. (**A**) Comparison of the number of animals with positive CSF cultures for *E. coli* between three groups receiving *E. coli* K1 WT, Δ*ybdO*, and *cybdO*. (**B**) Deletion of *ybdO* did not affect the magnitude of *E. coli* K1 bacteremia in mice. Bacterial counts in the blood (log CFU/mL) were determined 4 h after intravenous injection of *E. coli* K1 strains via tail vein. Data were analyzed using (**A**) a two-sided Fisher’s exact test and (**B**) Student’s *t*-test. * *p* < 0.05.

Because *E. coli* K1 invasion of HBMECs is associated with crossing the BBB, we investigated the contribution of YbdO to *E. coli* K1 pathogenesis by infecting mice with high-level bacteremia and examining the CSF cultures for bacterial meningitis. CSF samples were collected and cultured to illustrate the onset of bacterial meningitis. Induction of meningitis by the Δ*ybdO* strain resulted in a significantly reduced rate of meningitis occurrence (54.17%; *n* = 24) relative to that induced by the WT strain (84.62%, *n* = 26) (*p* = 0.0334) (Figure 3A). By contrast, the rate of meningitis occurrence induced by the *cybdO* strain (76.92%, *n* = 26) was comparable to that induced by the WT strain (Figure 3A), indicating that YbdO promotes meningitis in mice. Because different levels of bacteremia affect the percentage of meningitis occurrence, we further investigated whether *ybdO* deletion affected the level of bacteremia. The Δ*ybdO* mutant and WT induced similar levels of bacteremia (WT, 7.89 ± 0.04 mean log CFU/mL of blood vs. Δ*ybdO*, 7.54 ± 0.31 mean log CFU/mL of blood) (Figure 3B), suggesting that YbdO has no effect on *E. coli* K1 replication in mouse blood. Accordingly, we found that *ybdO* expression in mouse blood did not change significantly relative to that of the mutant grown in BHI medium (Figure S3). Collectively, these results suggested that YbdO promotes BBB penetration by *E. coli* K1 to cause meningitis in mice.

### 2.4. YbdO Promotes HBMEC Invasion by E. coli K1 by Enhancing Capsule Production

**Figure 4:**
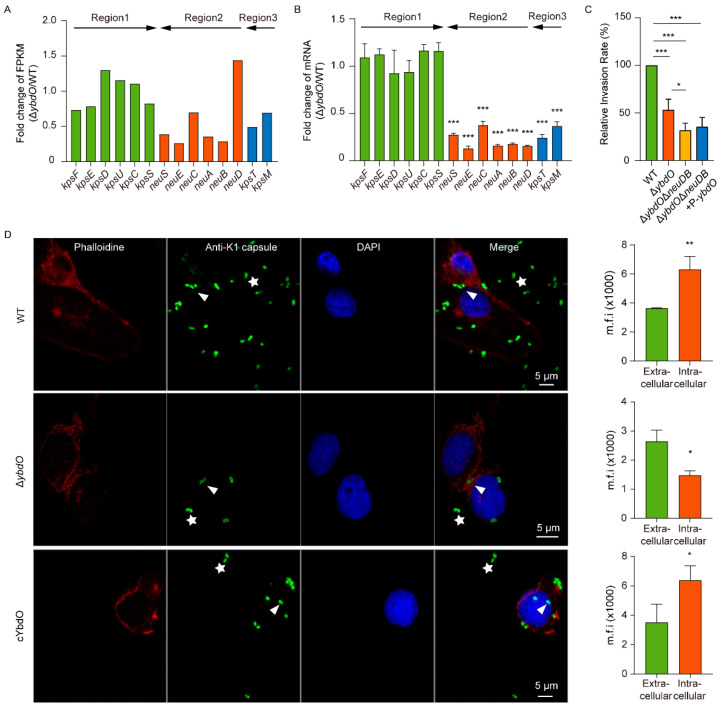
YbdO influences virulence by enhancing capsule production. (**A**) Altered transcription levels of K1 capsule genes following *ybdO* deletion. (**B**) Verification of the transcriptome data via qRT-PCR analysis of regions1, 2, and 3 in the K1 capsule. (**C**) Invasion rate of WT, Δ*ybdO*, Δ*ybdO*Δ*neuDB*, and Δ*ybdO*Δ*neuDB+P-ydbO E. coli* K1 in HBMECs. The invasion abilities of the deletion strains were calculated relative to that of the WT strain. (**D**) Invasion by the WT, Δ*ybdO*, and *cybdO E. coli* K1 strains according to immunofluorescence analysis of HBMECs. The actin cytoskeleton (red), K1 capsule (green), and nuclei (blue) are shown, intracellular bacteria are indicated by arrowheads, and extracellular bacteria are indicated by stars. Mean fluorescence intensity (m.f.i.) ± SD per bacterium per field in the intracellular (orange bars, *n* = 10) and extracellular samples (green bars, *n* = 10). (**B**–**D**) Data were obtained from three independent experiments and analyzed using Student’s *t*-test. * *p* < 0.05, ** *p* < 0.01, *** *p* < 0.001.

To further investigate the mechanism of YbdO-mediated promotion of HBMEC invasion by *E. coli* K1, we performed RNA-seq analysis using the WT and Δ*ybdO* strains to determine the downstream target genes of YbdO. We identified upregulated and downregulated expression of 58 and 108 genes, respectively, in the Δ*ybdO* mutant (Table S4). KEGG analysis revealed that these differentially expressed genes (DEGs) have diverse functions (Figure S4). Of particular relevance to *E. coli* K1 pathogenesis is the expression of several K1 capsule genes, including *kpsT*, which encodes ABC transporters (K1 capsule region 3), and *neuSEAB*, which is involved in O antigen nucleotide sugar biosynthesis (K1 capsule region 2), was downregulated by *ybdO* deletion, whereas the expression of genes of K1 capsule region 1 were not significantly affected (Figure 4A). This suggested that YbdO regulates the expression of genes of capsule regions 2 and 3 but not those of region 1. This result was further confirmed using qRT-PCR analysis (Figure 4B), the results of which correlated well with RNA-seq data and confirmed the validity of the latter. We identified no other known *E. coli* K1 virulence genes regulated by YbdO based on RNA-seq analysis (Table S4). The positive effect of YbdO on the expression of K1 capsule genes suggested that YbdO might promote *E. coli* K1 pathogenesis by increasing K1 capsule production.

Deletion of *ybdO* resulted in decreased expression of genes of regions 2 and 3, resulting in a decreased *E. coli* K1-invasion rate. To further verify whether YbdO contributes to *E. coli* K1 invasion and virulence by activating K1 capsule gene expression, we constructed a *ybdO*/*neuDB* double mutant strain (Δ*ybdO*Δ*neuDB*) and a complementary strain (Δ*ybdO*Δ*neuDB* strain complemented with *ybdO*, Δ*ybdO*Δ*neuDB+*P-*ybdO*) and performed HBMEC-invasion assays. The rate of HBMEC invasion by Δ*ybdO*Δ*neuDB* (31.95%) was lower than that by Δ*ybdO* (53.33%, *p* < 0.05), indicating that deletion of *neuDB* in Δ*ybdO* further decreased the invasion ability of the strain (Figure 4C). Additionally, the rate of HBMEC invasion by Δ*ybdO*Δ*neuDB+*P-*ydbO* (35.55%) was comparable with that by Δ*ybdO*Δ*neuDB* (Figure 4C), indicating that the K1 capsule plays an important role in the process of *E. coli* K1 invasion of HBMECs. These results confirmed that YbdO enhances *E. coli* K1 invasion by activating K1 capsule gene expression.

To investigate whether YbdO directly enhances capsule production, we performed immunofluorescence microscopy of the K1 capsule using anti-sialic acid, with capsule production quantified based on the fluorescence intensity of the fluorescein isothiocyanate (FITC)-labeled K1 capsule [39]. The fluorescence intensity of the FITC-labeled K1 capsule produced by Δ*ybdO* cultured in BHI medium did not differ significantly from that of the WT and *ybdO*-overexpressing strains (Figure S5), indicating that YbdO did not increase capsule production in culture medium. To further investigate the effect of YbdO on K1 capsule production during HBMEC invasion by *E. coli* K1, we stained the K1 capsule following *E. coli* K1 invasion of HBMECs with an immunofluorescent. Subsequent immunofluorescence microscopy revealed a 1.51-fold reduction in the fluorescence intensity of the capsule produced by the Δ*ybdO* mutant strain following HBMEC invasion as compared with that of the mutant before invading the HBMECs. By contrast, the fluorescence intensity of the capsule produced by the WT and c*ybdO* strains increased 1.74- and 1.81-fold, respectively, after HBMEC invasion as compared with that of the strains before invading the HBMECS (Figure 4D), indicating that YbdO is required for *E. coli* K1 production of K1 capsules in host cells.

**Figure 5:**
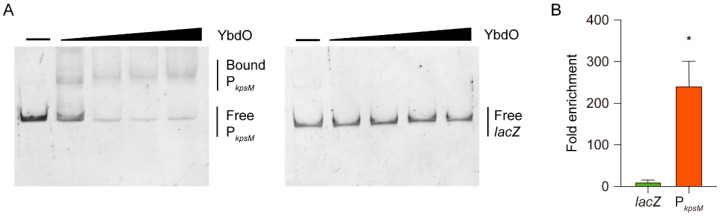
YbdO directly activates *kpsM* expression. (**A**) EMSA of the specific binding of YbdO to P_kpsM_ and *lacZ* (negative control). PCR products were added to the binding buffer at 20 ng each, and YbdO protein was added to the reaction buffer (lanes 1–5) at 0, 0.15, 0.3, 0.6, and 1.2 μM. (**B**) Fold enrichment of P_kpsM_ and *lacZ* in YbdO-ChIP samples. The fold enrichment of the YbdO-binding sites was measured based on the percent input of IP as compared with that of MOCK. Data were obtained from three independent experiments and analyzed using Student’s *t*-test. * *p* < 0.05.

To evaluate whether YbdO directly or indirectly regulates the expression of regions 2 and 3 genes, we performed electrophoretic mobility shift assays (EMSAs) using purified 6×-His-tagged YbdO and the potential promoter region of region 3 (300-bp upstream of the initial gene *kpsM*). The results showed that YbdO specifically binds to the *kpsM* promoter (Figure 5A), whereas no binding between YbdO and the *lacZ* fragment (used as a negative control) was detected under the same conditions (Figure 5A). Additionally, chromatin immunoprecipitation–quantitative PCR (ChIP-qPCR) analysis showed that the *kpsM* promoters were exceedingly enriched in the YbdO-ChIP samples, with their relative quantity in these samples 241-fold higher than that in the mock-ChIP control samples (Figure 2B). By contrast, the fold enrichment of *lacZ* (negative control) did not significantly differ between the YbdO-ChIP and mock-ChIP samples (Figure 5B). These results demonstrated that YbdO directly binds to the promoter region of the *kpsM* gene both in vitro and in vivo, suggesting that YbdO directly activates the expression of regions 2 and 3 genes by directly binding to the *kpsM* promoter.

### 2.5. H-NS Represses ybdO Gene Expression by Directly Binding to the ybdO Promoter

**Figure 6:**
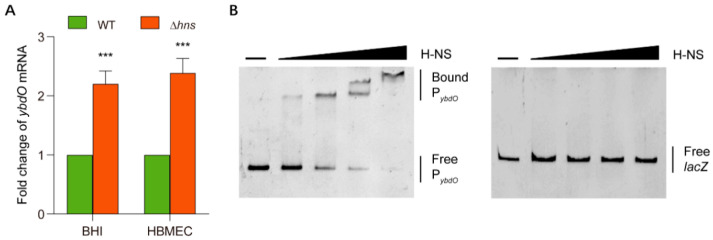
H-NS directly repressed *ybdO* expression. (**A**) Fold change in *ybdO* mRNA levels in the WT and Δ*hns* strains. (**B**) EMSAs of the specific binding of H-NS to P*_ybdO_* and *lacZ* (negative control). PCR products were added to the binding buffer at 20 ng each, and H-NS protein was added to the reaction buffer (lanes 1–5) at 0, 0.125, 0.25, 0.5, and 1 μM. Data were obtained from three independent experiments and analyzed using Student’s *t*-test. *** *p* < 0.001.

*ybdO* and other horizontally-transferred genes are under the control of the global regulator H-NS in *E. coli* K12 [36]. Therefore, we compared the homology of the *ybdO* promoter between *E. coli* K12 and K1 and found similarities in their high-AT ratio regions (Figure S6). To investigate whether H-NS regulates *ybdO* expression in *E. coli* K1, we constructed an Δ*hns* mutant strain and performed qRT-PCR to determine changes in *ybdO* expression. Deletion of *hns* in *E. coli* K1 resulted in 2.2- and 2.4-fold increases in *ybdO* expression in strains grown in BHI medium and after HBMEC invasion, respectively, relative to that observed in the WT strain (Figure 6A). These results indicated that H-NS was able to repress *ybdO* expression in *E. coli* K1. To evaluate whether H-NS directly represses *ybdO* expression, we performed EMSAs using purified 6×-His-tagged H-NS and the promoter region of *ybdO* (corresponding to 300 bp upstream). The results showed that H-NS binds specifically to the promoter region of *ybdO* in vitro (Figure 6B) but did not bind the *lacZ* fragment (negative control) [40] under the same experimental conditions (Figure 6B). These results indicated that H-NS represses *ybdO* gene expression by directly binding to the *ybdO* promoter.

### 2.6. Acidic pH Is a Host Cue to Induce ybdO Expression by Reducing H-NS Repression

**Figure 7:**
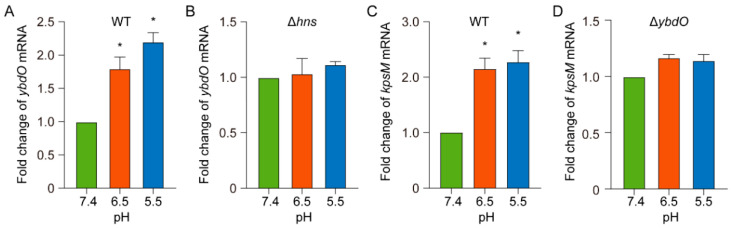
Acidic pH induces ybdO and kpsM expression. (**A**,**B**) Fold change in *ybdO* mRNA levels in the WT and Δ*hns* strains at various pH values. (**C**,**D**) Fold change in *kpsM* levels in the WT or Δ*ybdO* strains at various pH values. Data represent the means ± SD from three independent experiments and were analyzed using Student’s *t*-test. * *p* < 0.05.

We then investigated possible host signals that contribute to the induction of *ybdO* expression. H-NS release from targeted DNA-binding regions is reportedly induced by an acidic pH [41,42], which is a typical feature of early and late endosomes (pH ~ 6.5 and ~5.5, respectively) [43]. Notably, *E. coli* K1 traverses HBMECs through transcytosis via early and late endosomes [8]. To determine whether an acidic pH affects *ybdO* and *kpsM* expression, we simulated normal blood [44] and early and late endosomes using M9 medium at pH 7.4, 6.5, and 5.5. qRT-PCR results revealed 1.8- and 2.3-fold increases in *ybdO* expression upon exposure of the WT strain to pH 6.5 and pH 5.5, respectively, as compared with expression observed at pH 7.4 (Figure 7A). These results indicated that an acidic pH promotes *ybdO* expression. Furthermore, *ybdO* expression was not induced by an acidic pH in the Δ*hns* mutant strains (Figure 7B), indicating that an acidic pH induces *ybdO* expression via H-NS release from the *ybdO* promoter region.

Similar to *ybdO* expression, we observed 2.14- and 2.27-fold increases in *kpsM* transcript levels in the WT strain at pH 6.5 and pH 5.5, respectively, relative to those at pH 7.4 (Figure 7C). This suggested that *E. coli* K1 might increase capsule production to resist acidic conditions. Notably, activation of *kpsM* expression by an acidic pH was abolished by *ybdO* deletion (Figure 7D), indicating that an acidic pH promotes *kpsM* expression through YbdO. Collectively, these results suggested that acidic conditions in endosomes are a host signal sensed by *E. coli* K1 to reverse the H-NS-mediated repression of *ybdO* expression and thereby enhance capsule gene expression.

## 3. Discussion

Pathogens often exploit cunning strategies to adapt to the niche of the host. *E. coli* K1 is a critical common pathogen responsible for high morbidity and mortality in infants [1]; however, the mechanisms involved in regulating *E. coli* K1 virulence remain poorly understood. Here, we demonstrated that the transcriptional regulator YbdO promoted *E. coli* K1 invasion of HBMECs and meningitis in mice by directly activating the expression of K1 capsule region 2 and 3 genes by binding to the *kpsM* promoter. Additionally, *ybdO* expression was directly repressed by H-NS, with this inhibitory effect abolished under acidic conditions similar to those found in endosomes. Therefore, these findings identified a relationship between YbdO and both acidic pH and K1 capsule regulation.

**Figure 8:**
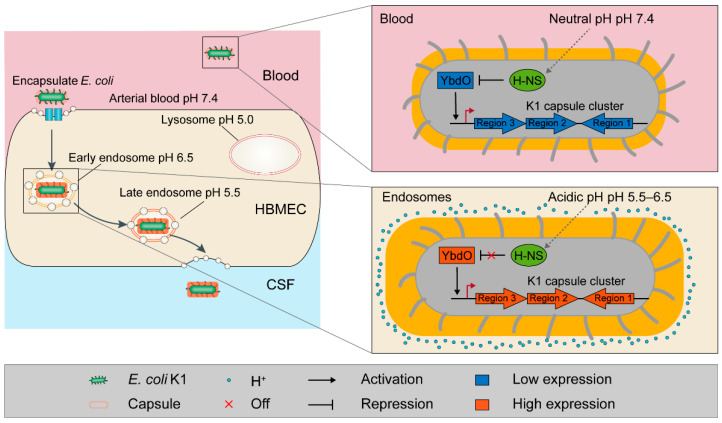
Model of the regulatory pathway of YbdO-mediated signaling in *E. coli* K1. In the blood, the pH is neutral (7.4), and H-NS binds to the *ybdO* promoter and represses *ybdO* expression, thereby inhibiting activation of capsule gene expression. In the endosomes of HBMECs, the pH is acidic (5.5–6.5), which induces H-NS detachment from the promoter to activate *ybdO* expression, allowing subsequent activation of capsule gene expression by YbdO to promote capsule production.

The ability of *E. coli* K1 to biosynthesize and assemble capsular polysaccharides is conferred by the K1 capsule genes [29,30]. Previous studies show that upregulation of capsular genes is often accompanied by increased capsular production [34,45,46]. In this study, we found that acidic pH induced capsule gene expression in the *E. coli* K1 WT strain (Figure 7C). It is reasonable to assume that acidic pH would increase the production of K1 capsular polysaccharides, although this requires further experimental confirmation. Therefore, we proposed a model of the YbdO-dependent K1 capsule regulatory pathway (Figure 8). Briefly, when *E. coli* K1 enters endosomes, the acidic pH relieves the H-NS-mediated repression of *ybdO* expression, after which activated *ybdO* expression promotes upregulation of K1 capsule gene expression, thereby increasing K1 capsule production to counteract the unfavorable environment in endosomes.

Previous transcriptome analysis of *E. coli* K1 bound to HBMECs detected a total of 227 genes that were differentially expressed in *E. coli* K1 associated with HBMEC as compared with expression observed in non-associated bacteria in the supernatant [47]. These genes are mainly involved in the presentation of cell-surface molecules, cellular function, and nitrogen metabolism. We constructed the transcriptome of *E. coli* K1 following its invasion of HBMECs and detected a total of 2700 DEGs relative to expression observed in bacteria cultured in BHI medium. These results demonstrated that *E. coli* K1 invasion of HBMECs is a complex process involving the expression of numerous genes, thereby offering insight into the mechanisms associated with *E. coli* K1 interactions with relevant target tissues. Complete elucidation of these transcriptional changes will provide additional information concerning *E. coli* K1–HBMEC interactions that are critical to understanding the pathogenesis of *E. coli* meningitis.

Our RNA-seq results showed that 166 regulator genes were differentially expressed (58 upregulated and 108 downregulated) in the Δ*ybdO* mutant as compared with the WT strain (Table S4), indicating YbdO as a global transcriptional regulator. In addition to K1 capsule genes, we found that *ybdO* deletion resulted in the downregulated expression of many known *E. coli* virulence genes, including *ompF* (encoding outer membrane porin F), *dnaJ* (encoding chaperone protein DnaJ), and *ibpB* (encoding the small heat-shock protein IbpB) (Table S4). Therefore, YbdO may exert its regulatory functions on *E. coli* K1 virulence and pathogenesis by affecting the expression of these virulence genes; however, confirmation of this will require further experimental studies.

RNA-seq analysis of genes exhibiting low expression can lead to relatively large fold biases and inaccurate results [48]. To confirm the RNA-seq results, we performed qRT-PCR analysis to validate the changes in expression of 14 capsule genes. For most of the genes examined, the fold change detected by real-time PCR was higher than that detected by RNA-seq. This was understandable, given that RNA-seq is generally less sensitive than qRT-PCR for quantifying gene expression [49,50]. Additionally, we found that some RNA-seq results were inconsistent with qRT-PCR results, especially those involving changes in *neuD* expression (Figure 4A,B). This could be because transcription levels of *neuD* in both WT and the Δ*ybdO* mutant were extremely low based on read counts (12 in WT and 15 in Δ*ybdO*) from RNA-seq results.

The typical expression of capsules in *E. coli* is regulated by temperature [34], whereas genes involved in capsule polysaccharide biosynthesis are upregulated in response to low oxygen and low iron levels [31]. We found that *E. coli* regulated the expression of the K1 capsule in response to an acidic environment in HBMECs. Acidic pH causes hydrolysis of the K1 capsule for capsule sloughing. Bacteria that have already produced polysialic acid (PSA) during growth under different conditions release their preformed PSA when placed in an acidic environment, such as in a phagolysosome [51,52,53]. Specifically, the Kl capsule produced in cultures at pH 7.0 was optimally released at pH 5.0. Therefore, it appears that K1 capsule production is increased in *E. coli* K1 to resist the acidic environment of endosomes and escape to fuse with lysosomes for survival and crossing of the BBB; however, the precise mechanisms involved in this process remain unclear and merit further investigation.

The K1 capsule increases the recovery of viable intracellular *E. coli* but attenuates binding to and internalization in HBMECs [8]. Therefore, the capsule is a double-edged sword for pathogens, and the spatial and temporal regulation of capsule production is of great significance for virulence. A previous study reported that bacterial adhesins shielded by the capsule also affect the interaction of *Neisseria meningitidis* with epithelial and endothelial cells, and that downregulation of capsule gene expression and removal of the sialic acid from the capsule are necessary for meningococcal interactions with host cells [54]. During urinary tract infection, uropathogenic *E. coli* downregulates the expression of capsule genes in urine for optimal adhesion to epithelial cells and switches from low or no capsule expression to increased capsule expression upon invasion of epithelial cells to allow the formation of intracellular bacterial communities [55]. In the present study, we found that *ybdO* deletion significantly decreased the capsule production and survival of intracellular *E. coli* K1 but had no obvious effect on *E. coli* K1 cultured in medium. We speculated that *E. coli* K1 expresses normal or low K1 capsule extracellularly to efficiently bind and invade HBMECs, followed by activation of the expression of K1 capsule genes by YbdO intracellularly to resist acidic challenges.

In conclusion, these findings enhance our understanding of how *E. coli* K1 utilizes environmental cues to facilitate HBMECs invasion and provide a paradigm for environmental signal sensing and virulence regulation that can be used to study other human bacterial pathogens.

## 4. Materials and Methods

### 4.1. Bacterial Strains, Plasmids, and Growth Conditions

The bacterial strains and plasmids used in this study are listed in Supplementary Table S1. The oligonucleotide primers used in this study are listed in Supplementary Table S2. *E. coli* K1 RS218 was used as the WT strain. Mutant strains were generated using the λ Red recombinase system of pSim6 and primers carrying the 50 bp homologous regions flanking the start and stop codons of the gene to be deleted, as previously described [56]. Bacteria were generally grown in BHI media at 37 °C; however, strains containing the temperature-sensitive plasmid pSim6 were cultured at 30 °C. For qRT-PCR analysis, bacteria were grown overnight in BHI media and inoculated at 1:100 into fresh M9 medium at different pH values (5.5–7.4; 37 °C; 200 rpm) until reaching the stationary phase [41]. The working concentrations of the antibiotics ampicillin, kanamycin, and chloramphenicol were 100 μg/mL, 50 μg/mL, and 25 μg/mL, respectively. All bacterial strains were frozen at −80 °C using 20% (v/v) glycerol.

### 4.2. E. coli Binding and Invasion Assays with HBMECs

HBMECs were a generous gift from Dr. K. S. Kim (Johns Hopkins University, Baltimore, MD, USA) and cultured in Roswell Park Memorial Institute (RPMI)-1640 medium with 10% fetal bovine serum (FBS), 10% Nu-serum, 2 mM glutamine, 1% MEM nonessential amino acids, 1 × MEM vitamin, 100 U/mL penicillin, 100 μg/mL streptomycin, and 1 mM sodium pyruvate. The *E. coli* strain was grown to the exponential phase at an optical density of 600 nm (OD_600_) of 0.6, collected via centrifugation, and resuspended in RPMI-1640 medium containing 10% FBS. HBMECs infected with a multiplicity of infection (MOI) of 100 were incubated at 37 °C in a 5% CO_2_ incubator for 90 min. The monolayers were then washed with warm phosphate-buffered saline (PBS) and incubated with experimental medium containing gentamicin (100 mg/mL) for 1 h at 37 °C to kill the extracellular *E. coli*. HBMECs were washed, lysed using 0.5% Triton X-100 in PBS, and cultured for determination of the CFUs. A binding assay was performed similar to the invasion assay, except with the omission of the gentamicin-treatment step [13].

### 4.3. Animal Model of E. coli Bacteremia and Hematogenous Meningitis

*E. coli* bacteremia and hematogenous meningitis were induced in BALB/c mice, which were ~14-days old (Vital River Laboratory Animal Technology Co., Beijing, China), as described previously [57]. All experiments were conducted according to protocols approved by the Institutional Animal Care Committee at Nankai University (Tianjin, China). Each mouse received *E. coli* (1 × 10^6^ CFU) in the exponential phase in 100 µL of PBS via tail vein injection. After 4 h, blood and CSF specimens were collected for determination of the CFUs and for RNA extraction. Meningitis was defined as a positive culture in CSF [58]. For determination of the CFUs, the bacteria in the blood specimens were subjected to serial 10-fold dilutions in PBS and enumerated by plating on BHI agar plates. For RNA extraction, the mice were sacrificed, and blood specimens were collected to extract RNA using TRIzol reagent (Invitrogen, Carlsbad, CA, USA).

### 4.4. qRT-PCR

qRT-PCR was performed using an ABI QuantStudio 5 real-time PCR system (Applied Biosystems, Foster City, CA, USA). The *E. coli* strains were cultured overnight and subsequently subcultured (1:100) in fresh BHI medium to the exponential phase. Bacteria were pelleted via centrifugation, and RNA samples were isolated using TRIzol (Invitrogen), reverse transcribed using a PrimeScript RT reagent kit (Takara, Shiga, Japan), and processed for qRT-PCR. Each qRT-PCR was performed using Power SYBR Green PCR master mix (Applied Biosystems). The fold change in the expression of the target gene relative to that of the housekeeping gene (*dnaE*) was determined using the 2^−ΔΔCt^ method [59]. At least three biological replicates were performed for each qRT-PCR analysis.

### 4.5. RNA-seq

*E. coli* K1 was collected in the exponential phase in BHI or during HBMEC invasion, and total RNA was isolated using TRIzol reagent (Invitrogen) according to manufacturer instructions. RNA was quantified and qualified using a Bioanalyzer 2100 (Agilent Technologies, Santa Clara, CA, USA), a NanoPhotometer spectrophotometer (Implen GmbH, Munich, Germany), and 1% agarose gel electrophoresis. For library preparation, we used 3 µg of total RNA per sample. rRNA was depleted from total RNA using a Ribo-off rRNA depletion kit (Vazyme, Nanjing, China), and libraries were constructed and analyzed by NOVOGENE, Inc. (TianJin, China). DEGs in HBMEC-invading *E. coli* K1 were identified using the DESeq R package. Available online: https://www.nuget.org/packages/PuppeteerSharp (accessed on 13 April 2022). Their expression was compared with that of BHI-cultivated *E. coli* K1. The resulting *p*-values were adjusted using the Benjamini–Hochberg test for controlling the false discovery rate. Genes with an adjusted *p* < 0.05 were considered as differentially expressed. The other transcriptomes of the WT and Δ*ybdO* mutant strains were processed and compared using the same methods.

### 4.6. ChIP-qPCR Analyses

For the FLAG-tagged plasmids, the coding DNA sequence was amplified from the RS218 genome using PCR and cloned into the pBAD24 plasmid to allow *ybdO* overexpression. The WT strain containing pBAD-YbdO was cultured in LB medium supplemented with ampicillin and 0.1% arabinose until the mid-log phase (OD_600_ = 0.6) and then treated with 1% formaldehyde for 10 min at 25 °C. Cross-linking was stopped by the addition of 125 mM glycine. Bacterial pellets were washed twice with PBS buffer, resuspended in immunoprecipitation buffer [IP, 50 mM, (pH 7.5), HEPES–KOH, 150 mM NaCl, 1 mM EDTA, 1% Triton X-100, 0.1% sodium deoxycholate, 0.1% SDS, and protease inhibitor cocktail (Medchem Express LLC, Monmouth Junction, NJ, USA)], and then subjected to sonication to produce 250 to 500 bp DNA fragments. Insoluble cellular debris was removed via centrifugation at 4 °C, and the supernatant was used as the input sample in the immunoprecipitation experiments. Both the mock and immunoprecipitated samples were separately incubated with isotype and anti-FLAG antibodies and then incubated with protein A beads in an immunoprecipitation buffer. Washing, cross-link reversal, and purification of the ChIP DNA were then conducted. To measure the enrichment of the potential YbdO-binding targets in the immunoprecipitated DNA samples, the percent input and fold enrichment were determined using SYBR green PCR master mix. The relative target levels were calculated using the ΔCt method, with *lacZ* used as a negative control according to a previous study [60]. The results were reported as the average enrichment of three biological replicates.

### 4.7. Immunofluorescence Assays

Immunofluorescence analysis was performed as described previously [39]. Briefly, bacteria were subcultured at a 1:100 ratio in BHI and incubated overnight at 37 °C with shaking at 180 rpm until an OD_600_ of 0.6 was obtained. The bacteria were then diluted on coverslips to allow HBMEC infection at an MOI of 100 in the exponential phase. After 2.5 h of incubation, the coverslips were washed and fixed with formaldehyde, and the cells were permeabilized with 0.1% Triton X and stained with fluorescein AF647-labeled phalloidin to visualize the actin filaments. *E. coli* was stained with the FITC-labeled anti-*E. coli* K and O antigen antibody (Abcam, Cambridge, UK). The K1 capsule was stained with an anti-PSA antibody and AF488-labeled secondary antibody (Abcam, Cambridge, UK). HBMEC nuclei were stained with 6-diamidino-2-phenylindol. Invasion assays were performed for each cell line, with three slides per experiment.

### 4.8. EMSAs

The 6×-His-tagged H-NS (N-terminus) and YbdO (C-terminus) proteins were expressed in *E. coli* BL21/DE3 containing pET-H-NS and pET-YbdO plasmids, respectively, and purified from soluble extracts using a Ni-NTA-chelating column (Thermo Scientific, Waltham, MA, USA), as previously described [41] Protein concentrations were determined using a bicinchoninic acid protein assay, and the proteins were aliquoted and stored at −80 °C. The PCR fragments containing the promoter regions of *kpsM* and *ybdO* and the *lacZ* fragment (negative control) [40] were amplified using the genomic DNA of the RS218 strain as a template. The fragments were then gel-purified, and 20 ng of the DNA fragments was incubated with purified protein (0–2 µM) in 20 µL of a solution containing band-shift buffer [10 mM Tris (pH 7.5), 1 mM EDTA, 100 mM KCl, 0.1 mM DTT, 5% (v/v) glycerol, and 0.01 mg/mL bovine serum albumin] [61] at 25 °C for 30 min. Native 8% (w/v) polyacrylamide gels were used to separate the samples in 0.5 × Tris-borate-EDTA, and the gels were then stained with GelRed (Genestar, Beijing, China).

### 4.9. Growth Assay

To determine the growth curve of each strain, overnight cultures were washed with PBS three times and diluted (1:1000) in BHI broth without antibiotics. A 200 μL aliquot was added to a 96-well flat-bottom microplate and incubated at 37 °C with shaking at 180 rpm for 24 h, as previously described [62]. The absorbance at 600 nm was recorded. Experiments were independently performed three times.

### 4.10. Statistical Analysis

Statistical analysis was conducted using GraphPad Prism software (v8.3.0; GraphPad Software, San Diego, CA, USA). The mean ± SD from three independent experiments is shown in the figures. Differences between two mean values were evaluated using a two-tailed Student’s *t*-test. Statistical significance was assessed using the two-sided Fisher’s exact test for the animal meningitis experiments. Statistical significance was set at a *p* < 0.05.

## Data Availability

The RNA-seq data acquired in this study are available in Sequence Read Archive (SRA) data repository (accession code: PRJNA822028, https://www.ncbi.nlm.nih.gov/bioproject/PRJNA822028, accessed on 13 April 2022). Other data are presented within manuscript and Supplementary Materials.

## Supplementary Materials

The following supporting information can be downloaded at: https://www.mdpi.com/article/10.3390/ijms23105543/s1.

## Abbreviations

HBMEC	Human brain microvascular endothelial cell
H-NS	Histone-like nucleoid structuring protein
CSF	Cerebrospinal fluid
BBB	Blood–brain barrier
CNF1	Cytotoxic necrotizing factor 1
OmpA	Outer membrane protein A
LTTR	Lysr-type transcriptional regulator
HTH	Helix-turn-helix
BHI	Brain–heart infusion
KEGG	Kyoto Encyclopedia of Genes and Genomes
TFs	Transcriptional regulators
qRT-PCR	Quantitative reverse transcription polymerase chain reaction
DEG	Differentially expressed gene
FITC	Fluorescein isothiocyanate
EMSA	Electrophoretic mobility shift assay
ChIP-qPCR	Chromatin immunoprecipitation-quantitative PCR
PSA	Polysialic acid
FBS	Fetal bovine serum
RPMI	Roswell Park Memorial Institute
MOI	Multiplicity of infection
PBS	Phosphate-buffered saline
OD_600_	Optical density at 600 nm
