# Supplementary material for: YbdO Promotes the Pathogenicity of Escherichia coli K1 by Regulating Capsule Synthesis
Source: Int J Mol Sci. 2022 May 16;23(10):5543. doi: 10.3390/ijms23105543 (PMC9141747; doi:10.3390/ijms23105543)
Supplement: Supplementary file 1 [file ijms-23-05543-s001.zip › ijms-1704989-supplementary/Supplimentary _Materials.pdf]

Supplementary materials

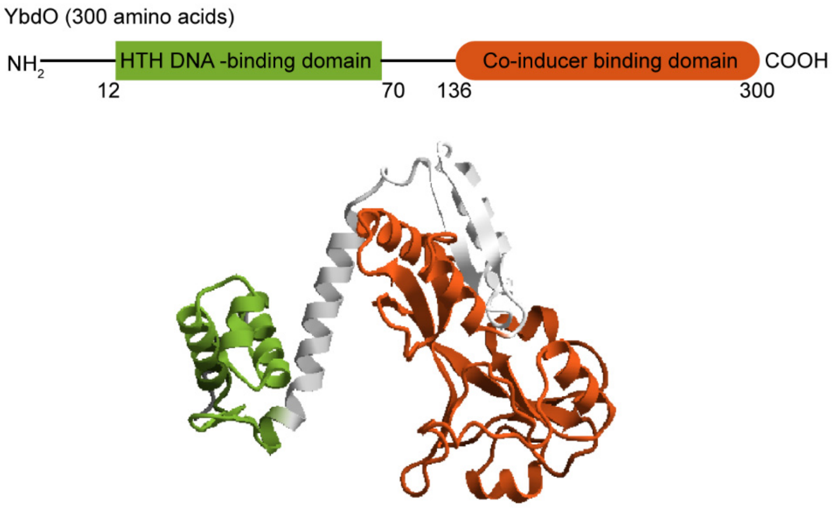

Figure S1. Three-dimensional representations of the HTH DNA-binding motif and the co-inducer-binding domain motif of YbdO.

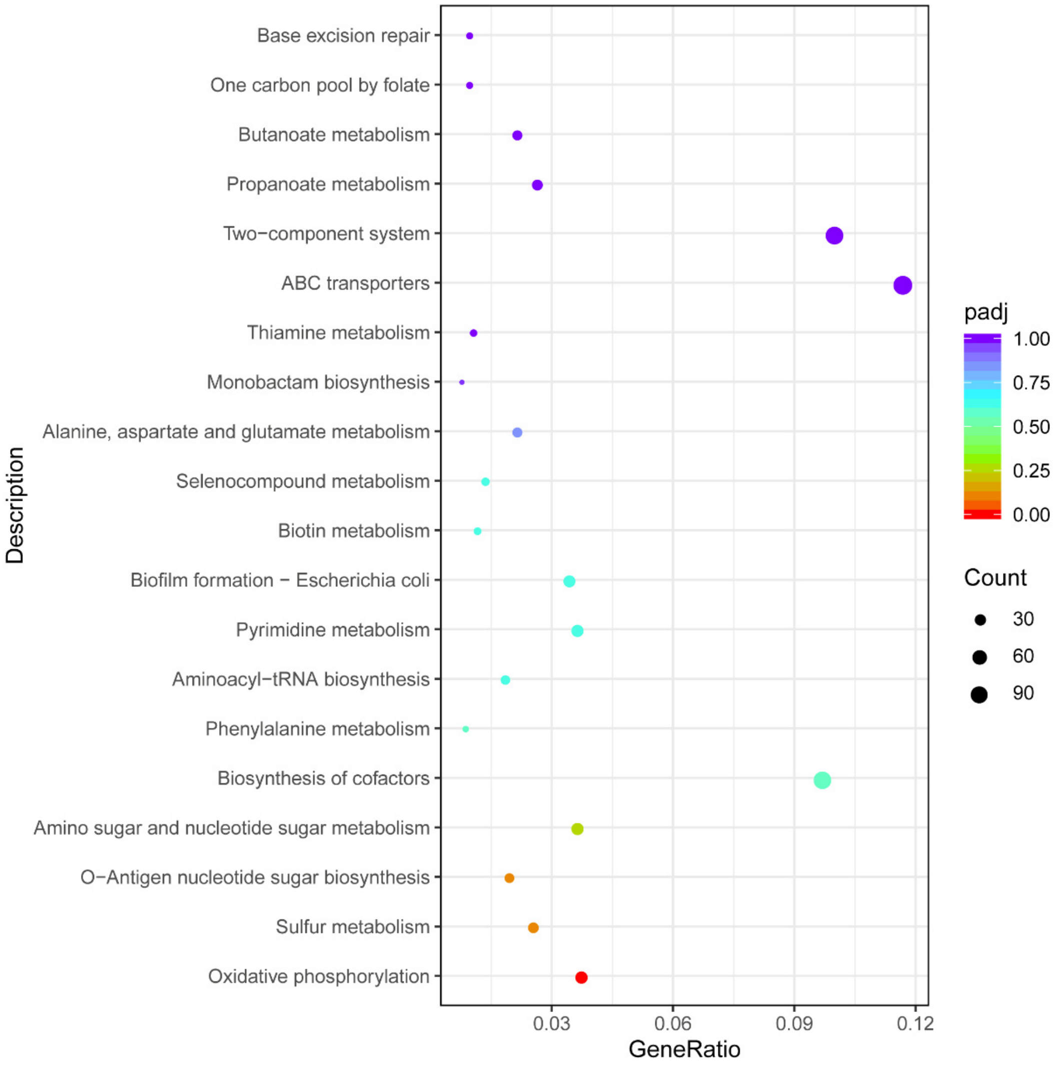

Figure S2. RNA-seq analysis of the genes involved in *E. coli* K1 invasion of HBMECs.

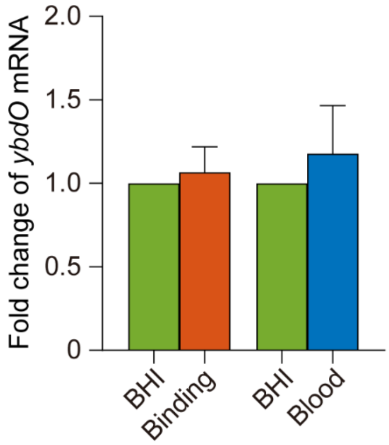

Figure S3. *ybdO* expression is unchanged upon *E. coli* K1 binding to HBMECs or invasion of blood.

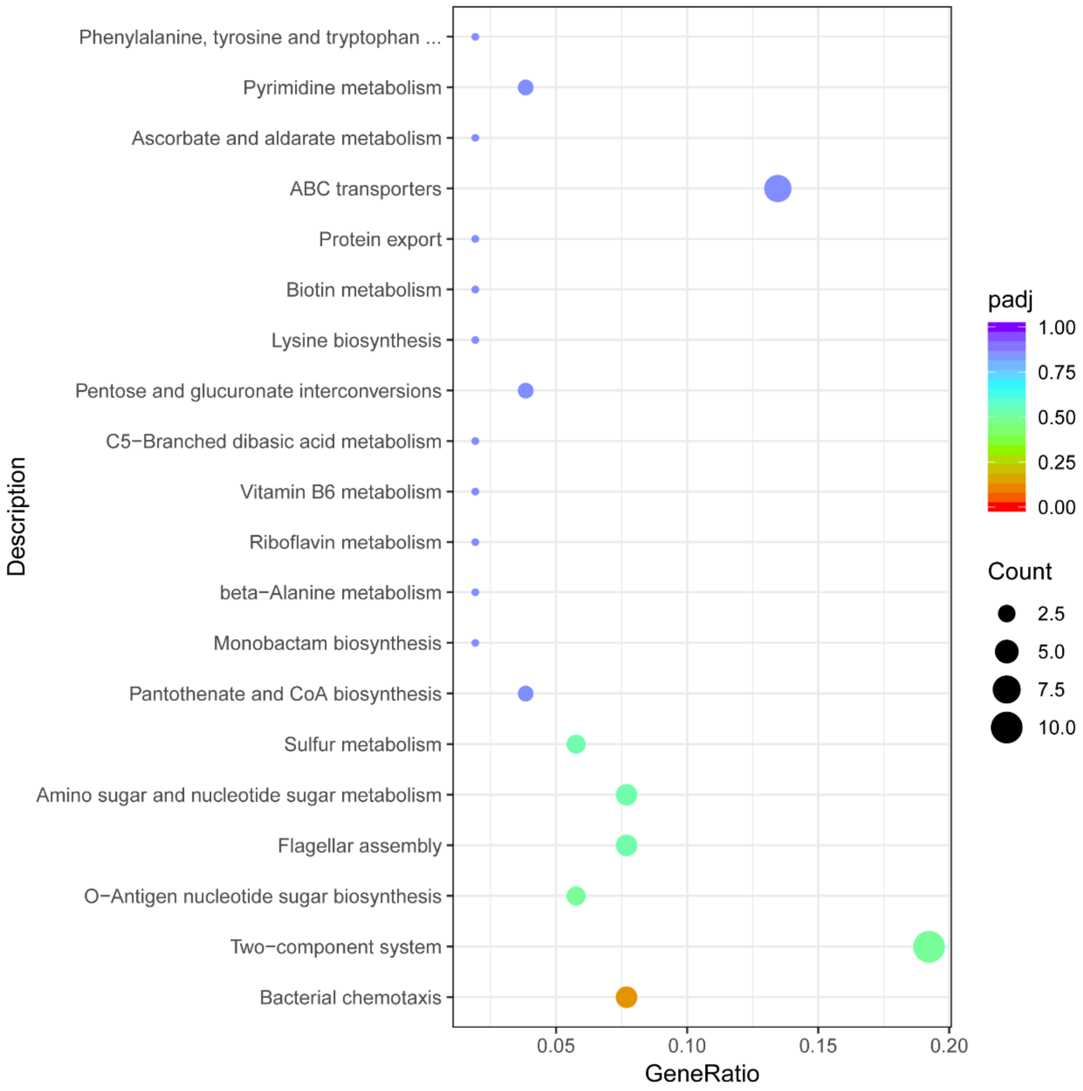

Figure S4. RNA-seq analysis of genes involved in the *ybdO*-mutation strain.

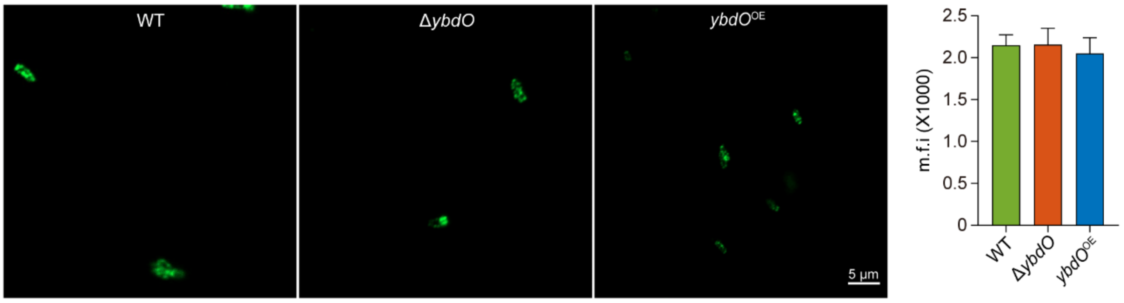

Figure S5. YbdO does not increase capsule production in culture medium. The production of the K1 capsule was measured via immunofluorescence staining. K1 capsule (green). Scale bar, 5 μm. Mean fluorescence intensity (m.f.i.) ± SD. per bacterium per field from the mutant strain Δ*ybdO* (orange bars, n = 10) and the overexpression strain *ybdO*^{OE} (blue bars, n = 10) compared to the WT (green bars, n = 10) samples. Data are presented as the means ± SD from three independent experiments.

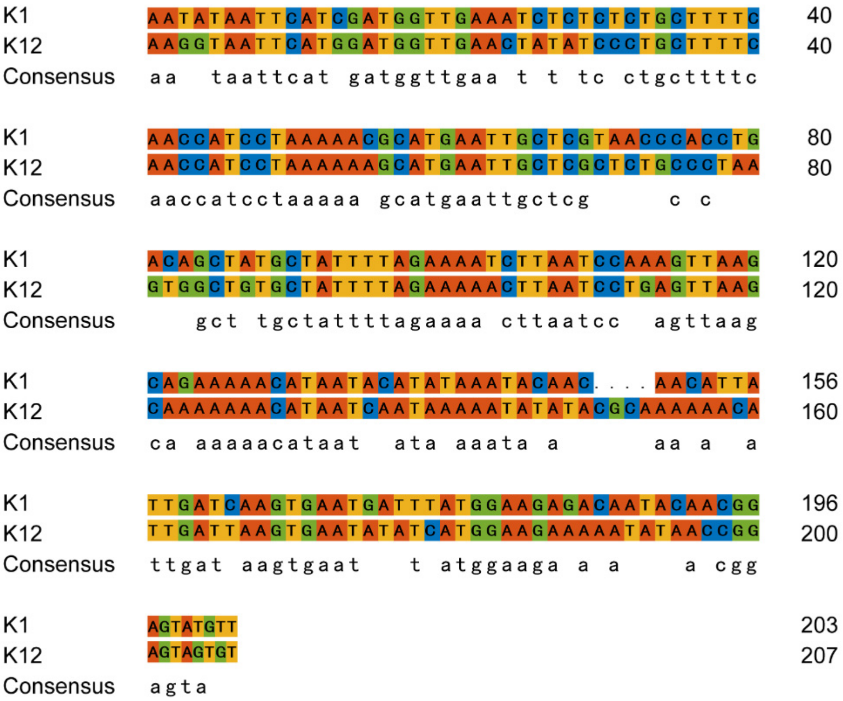

Figure S6. Two-sequence alignment of *ybdO* promoters from *E. coli* K1 and K12.

Supplementary Table S1. Bacterial strains and plasmids used in this study.

	Genotype or Description	Source
***E. coli* K1 strains**		
WT	Wild-type *E. coli* K1 RS218	Our lab
Δ*ybdO*	WT strain *ybdO*::Cm; Cm^R	This study
Δ*hns*	WT strain *hns*::Cm; Cm^R	This study
Δ*neuDB*	WT strain *neuDB*::Cm; Cm^R	This study
cYbdO	Δ*ybdO* harboring plasmid P-*ybdO*; Cm^R, Tc^R; complement strain	
Δ*ybdO*Δ*neuDB*	WT strain *ybdO*::Cm,*neuDB*::Km; Cm^R, Km^R	
Δ*ybdO*Δ*neuDB* +P-*ybdO*	Δ*ybdO*Δ*neuDB* containing plasmin P-*ybdO*; Cm^R, Km^R, Tc^R	
ybdO^{OE}	WT harboring plasmid pBAD-*ybdO*; Ap^R; overexpressing strain	This study
BL21	For expressing protein	TransGen Biotech
**Plasmids**		
pKD3	For λ Red recombination; Cm^R	Our lab
pSim6	For generating mutant strains with λ Red recombinase system under an temperature-inducible promoter; Ap^R	Our lab
pACYC184	A cloning vector; Cm^R, Tc^R	
P-*ybdO*	pACYC184 plasmid carrying the WT *ybdO* gene; Tc^R	
pBAD24	Overexpression vector; Ap^R	Our lab
pET28a	Expression vector; Km^R	Our lab
pET32a	Expression vector; Ap^R	Our lab
pET-*hns*	pET28a carrying the WT *hns* gene; Km^R	Our lab
pET-*ybdO*	pET32a carrying the WT *ybdO* gene; Ap^R	Our lab
pBAD-*ybdO*	pBAD24 carrying the WT *ybdO* gene; Ap^R	This study

Supplementary Table S2. Primers used in this study.

Target Gene		Primer Sequence (5'–3')
**Primers for construction of mutant***		
ybdO	F	ATGGCTAATCTTTATGATCTAAAAAAATTTGACCTCAATTTACTGGTCATCATATGAATATCCTCCTTAG
	R	AGTTCGGTGGGCACAATGCCTAATAAGTCAGTAACAGAAATACTATTAATGTGTAGGCTGGAGCTGCTTCG
hns	F	TCTATTATTACCTCAACAAACCACCCCAATATAAGTTTGAGATTACTACAGCCATGGTCCATATGAATATCCTCC
	R	AAAAAATCCCGCCGATGGCGGGATTTTTAGCATGTGCAATCTACAAAAGAGTGTAGGCTGGAGCTGCTTCG
neuDB	F	TGGTTACATTCCAATATTATGCCTTGGAAATATTTAACTGAGACATATCCATATGAATATCCTCCTTAG
	R	GAATTCGCTATGAATTATTAATTCATCTGGAATAAAGTCTTGCTCTGCAAGTGTAGGCTGGAGCTGCTTCG
**Primers for identification of mutant**		
ybdO	F	AAGTGTTAAGCGACAACGAG
	R	GCGCTTCCCAGTCGATAAAC
hns	F	TAGGGCTATATGCCGCGTC
	R	TGGCTTGAAGAAGAGATGGGC
neuDB	F	GGTGTCACATAGTGAACGAGC
	R	GCTCCATACTGTTCGGAGTCAG
**Primers for construction of clone and complemented strains**		
ybdO	F	CCTTGCGTATAATATTTGCCCATGGTTGGTTGGGGTTATCAAGCC
	R	CTGATGAATGCTCATCCGGAATTCTGTCAGTGGTAATGAATCAGAAGC
YbdO-Flag	F	TGGGCTAGCAGGAGGAATTCCGGAGTATGTTATGGCTAATCTTTATG
	R	CCGCCAAAACAGCCAAGCTTCTACTACTTGTCATCGTCATCCTTGTAATCGATGTCATGATCTTTATAATCACCGTCATGGTCTTTGTAGTCGAAGCTATCATTTAAACGATCTATGAATCG
H-NS-His	F	CAGCAAATGGGTCGCGGATCCATGAGCGAAGCACTTAAAATTCTG
	R	AGTGGTGGTGGTGGTGGTGCTCGAGTTGCTTGATCAGGAAATCGTCGAGG
YbdO-His	F	GACGACGACGACAAGGCCATGGCGGAGTATGTTATGGCTAATCTTTATG
	R	AGTGGTGGTGGTGGTGGTGCTCGAGGAAGCTATCATTTAAACGATCTATGAATCG
**Primers for qRT-PCR**		
ybdO	F	AGGATGTCATGGCATTCTAT
	R	CCGTTATTCATTCGCTCAG
dnaE	F	GGCAATCGTATCGGTATCT
	R	GCTTCGTCAATATCCATCAC
kpsM	F	AGTTCAGACTCACGCCTTCACT
	R	AGGTCCTGCCGATACTGCTTAA
kpsT	F	AATCCACCAGCCAATCCAACGG
	R	AAGTCTTATCGCACGCCCACTG
neuD	F	GGAGTCCCAGCAACTACAACAT
	R	AGGCTGCTGTAGCAATATCTCT
neuB	F	CCACTCCATCGGACTGATACCA
	R	TGGCCCAGATCATTTGGCTCAA
neuA	F	CTGTTCGGAGTCAGTTGTCACA
	R	TGCGATAATTCCAGCCCGTAGT
neuC	F	ATTACACTGGCTCCACGAACAC
	R	GTAACGCTGACACTGGTTCTGA
neuS	F	ACCATGAGTCACGGAAAAACCC
	R	TCCTGGCATTGTCCTTACTTCT
neuE	F	TTTAGGCTCTTTAGGGTGAGGTTT
	R	GGTGCTCCATCAGTTAAATCATCA
kpsF	F	ATGCTGATGCCGTGCTGGAAC

	R	GCAGGCGACGACCTAATGAACC
kpsE	F	AACCAGACCGTGCTGAAAGAGT
	R	CTGTGCCTGTGCCTGTGGAT
kpsD	F	CTGGTCTGTATGGCGGTGTGA
	R	GCCGTTCAGCAGGAAGTCGTA
kpsU	F	GCAGGGCGACGAACCAATGATT
	R	ACGGAATAGGCGAACGGCTGAA
kpsC	F	CGCCCTGTTCCGCAAGAAGTTA
	R	GCTCGCCATTGACGCCAAGAT
kpsS	F	ACTGCCGTCATCGACAATACCT
	R	TGCGTTCACTCCGTCTTCTTCA
**Primers for ChIP-qPCR**		
lacZ	F	CACCGCCGAAAGGCGCGG
	R	CAGCCGCGCGGTACTGGAG
kpsM	F	GCGGCTATTAAAAAGGTCAAACCG
	R	GCCATTTGATGATGTGATCCTAATCTC
**Primers for EMSAs**		
lacZ	F	GCGGCCCCAGGCCGAGCC
	R	CCTACCGGATTGATGGGCACGGTGAGATGG
P_{ybdO}	F	ACATACTCCGTTGTATTGTCTCTTCC
	R	TACCCTGCAACAAGCCGTC
P_{kpsM}	F	CCATTTGATGATGTGATCCTAATCTC
	R	TTTCCTGAGAAATTAACTCTGCATTC

* Primers were designed to harbor extensions homologous to ~50 bp (underlined) of the target gene; F, forward; R, reverse.
